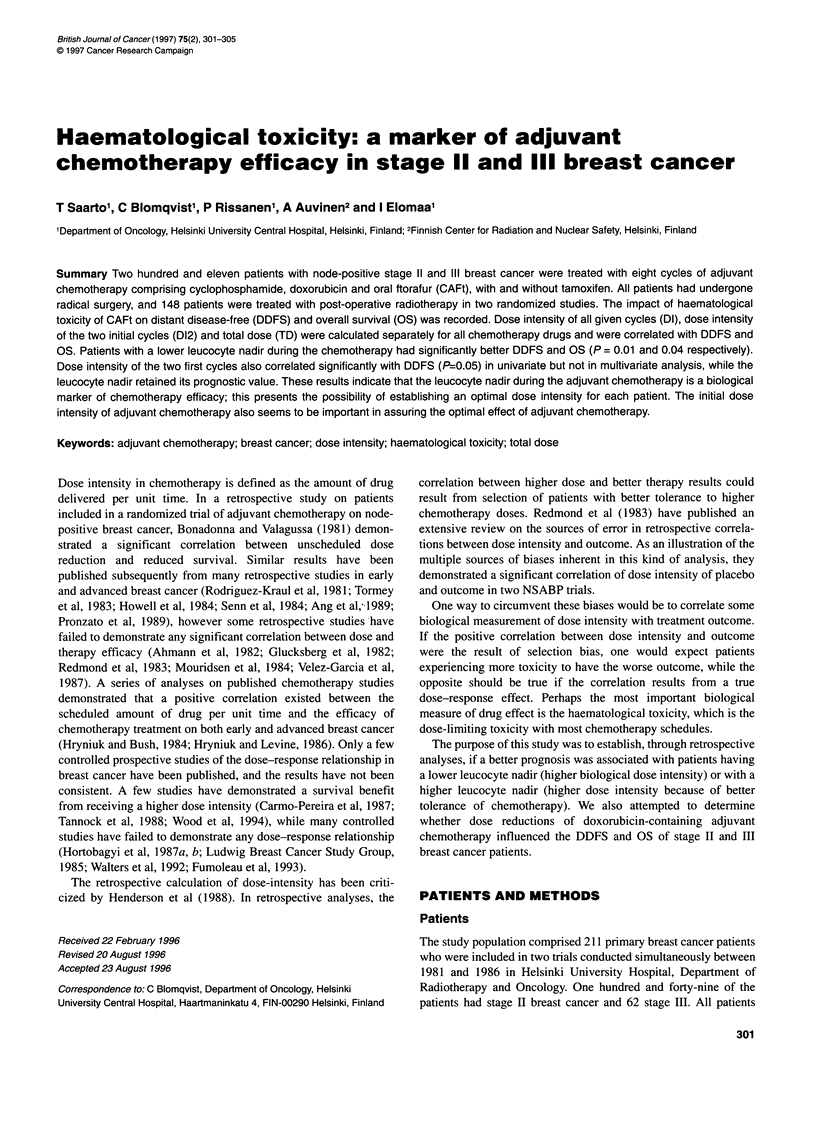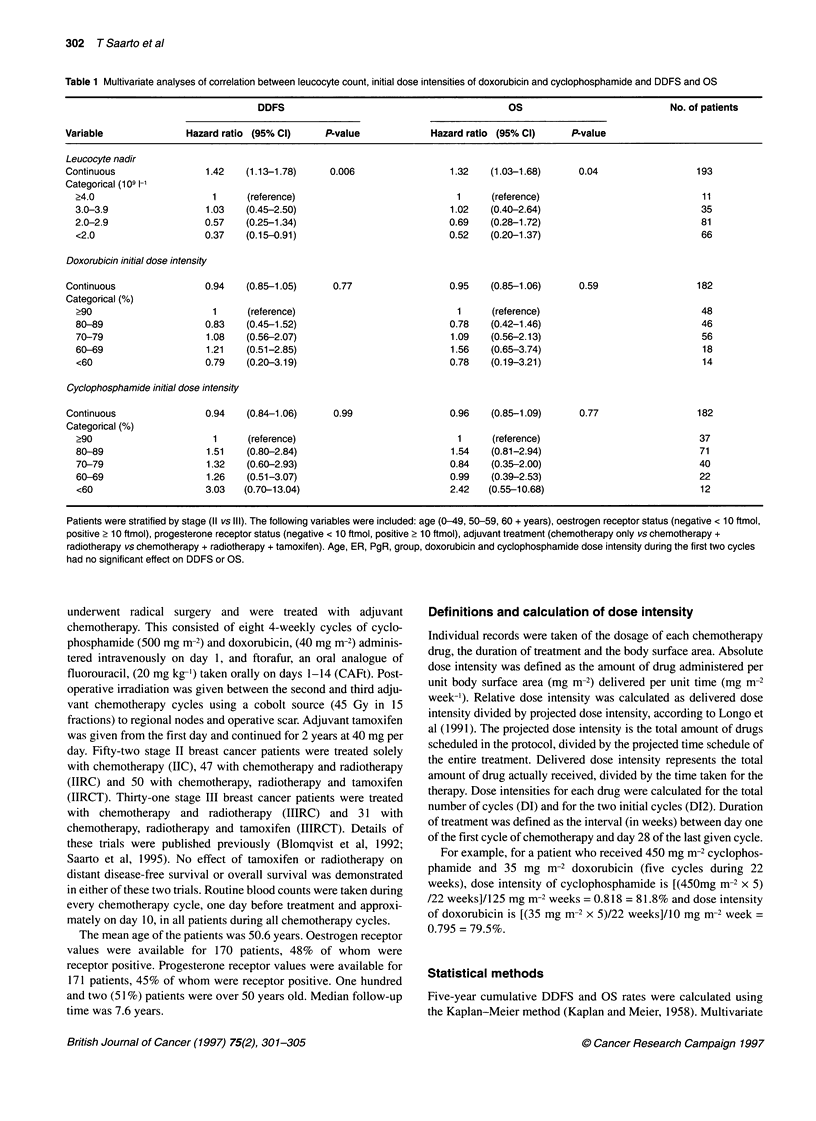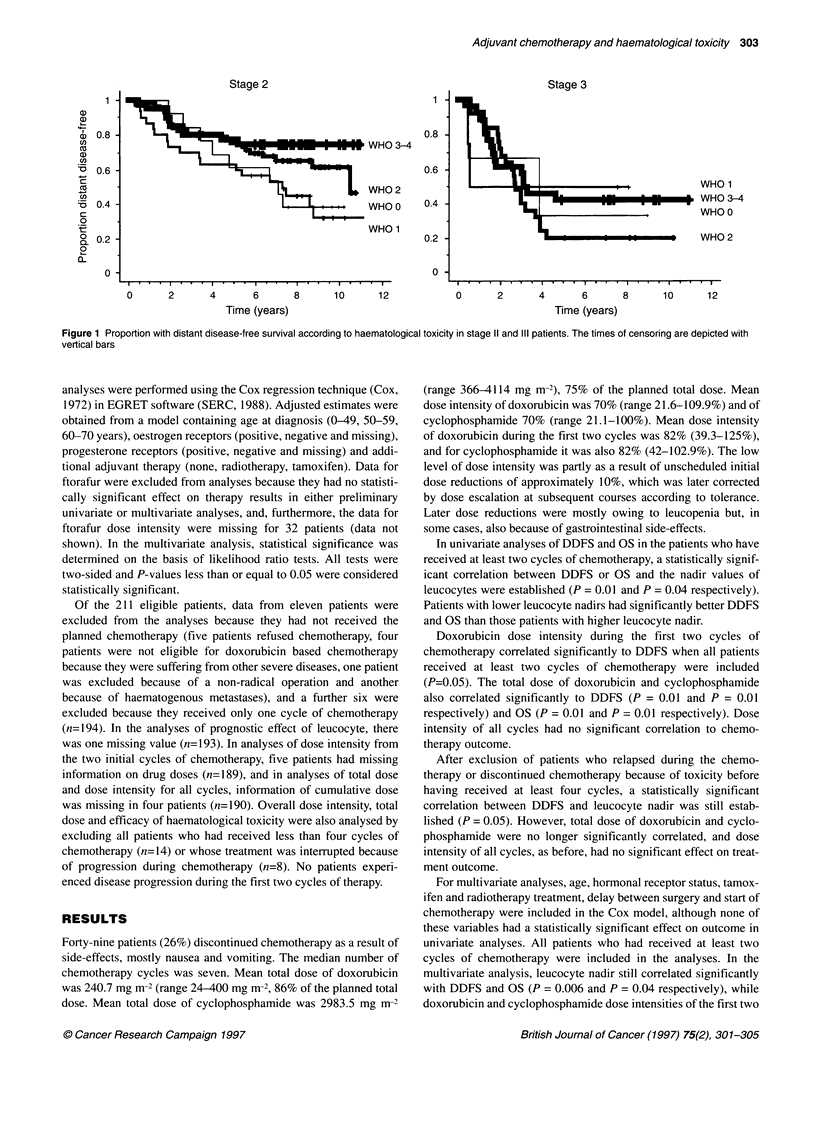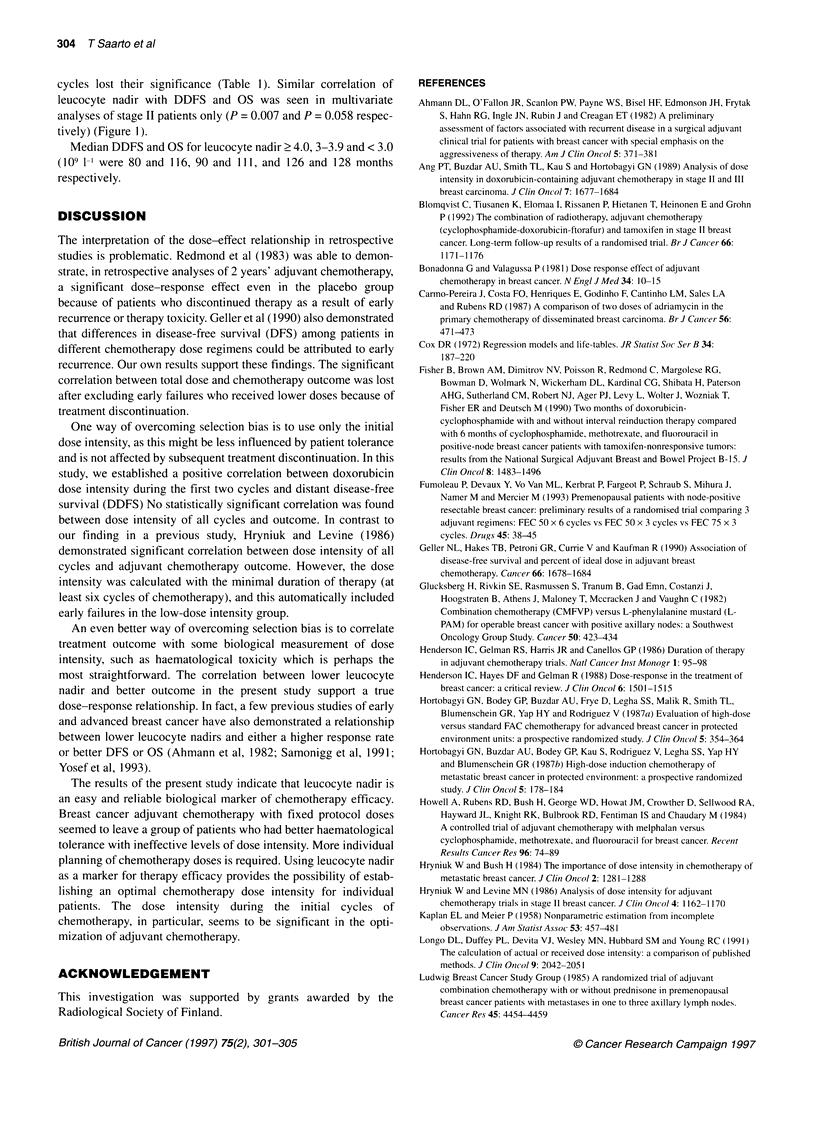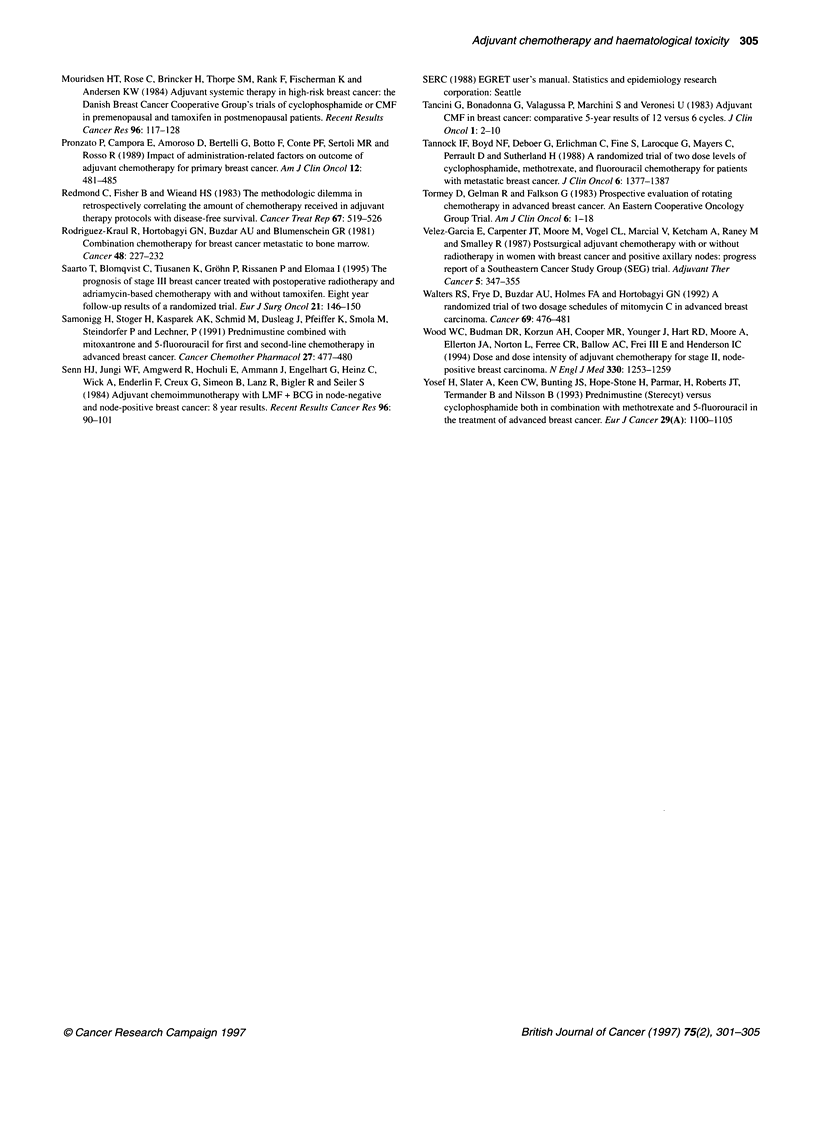# Haematological toxicity: a marker of adjuvant chemotherapy efficacy in stage II and III breast cancer.

**DOI:** 10.1038/bjc.1997.49

**Published:** 1997

**Authors:** T. Saarto, C. Blomqvist, P. Rissanen, A. Auvinen, I. Elomaa

**Affiliations:** Department of Oncology, Helsinki University Central Hospital, Finland.

## Abstract

Two hundred and eleven patients with node-positive stage II and III breast cancer were treated with eight cycles of adjuvant chemotherapy comprising cyclophosphamide, doxorubicin and oral ftorafur (CAFt), with and without tamoxifen. All patients had undergone radical surgery, and 148 patients were treated with post-operative radiotherapy in two randomized studies. The impact of haematological toxicity of CAFt on distant disease-free (DDFS) and overall survival (OS) was recorded. Dose intensity of all given cycles (DI), dose intensity of the two initial cycles (DI2) and total dose (TD) were calculated separately for all chemotherapy drugs and were correlated with DDFS and OS. Patients with a lower leucocyte nadir during the chemotherapy had significantly better DDFS and OS (P = 0.01 and 0.04 respectively). Dose intensity of the two first cycles also correlated significantly with DDFS (P = 0.05) in univariate but not in multivariate analysis, while the leucocyte nadir retained its prognostic value. These results indicate that the leucocyte nadir during the adjuvant chemotherapy is a biological marker of chemotherapy efficacy; this presents the possibility of establishing an optimal dose intensity for each patient. The initial dose intensity of adjuvant chemotherapy also seems to be important in assuring the optimal effect of adjuvant chemotherapy.


British Journal of Cancer (1997) 75(2), 301–305
© 1997 Cancer Research Campaign

### Haematological toxicity: a marker of adjuvant chemotherapy efficacy in stage II and III breast cancer

**T Saarto[1], C Blomqvist[1], P Rissanen[1], A Auvinen[2] and I Elomaa[1]**

[1]Department of Oncology, Helsinki University Central Hospital, Helsinki, Finland; [2]Finnish Center for Radiation and Nuclear Safety, Helsinki, Finland

**Summary** Two hundred and eleven patients with node-positive stage II and III breast cancer were treated with eight cycles of adjuvant chemotherapy comprising cyclophosphamide, doxorubicin and oral ftorafur (CAFt), with and without tamoxifen. All patients had undergone radical surgery, and 148 patients were treated with post-operative radiotherapy in two randomized studies. The impact of haematological toxicity of CAFt on distant disease-free (DDFS) and overall survival (OS) was recorded. Dose intensity of all given cycles (DI), dose intensity of the two initial cycles (DI2) and total dose (TD) were calculated separately for all chemotherapy drugs and were correlated with DDFS and OS. Patients with a lower leucocyte nadir during the chemotherapy had significantly better DDFS and OS ($P = 0.01$ and 0.04 respectively). Dose intensity of the two first cycles also correlated significantly with DDFS ($P$=0.05) in univariate but not in multivariate analysis, while the leucocyte nadir retained its prognostic value. These results indicate that the leucocyte nadir during the adjuvant chemotherapy is a biological marker of chemotherapy efficacy; this presents the possibility of establishing an optimal dose intensity for each patient. The initial dose intensity of adjuvant chemotherapy also seems to be important in assuring the optimal effect of adjuvant chemotherapy.

**Keywords:** adjuvant chemotherapy; breast cancer; dose intensity; haematological toxicity; total dose

Dose intensity in chemotherapy is defined as the amount of drug delivered per unit time. In a retrospective study on patients included in a randomized trial of adjuvant chemotherapy on node-positive breast cancer, Bonadonna and Valagussa (1981) demonstrated a significant correlation between unscheduled dose reduction and reduced survival. Similar results have been published subsequently from many retrospective studies in early and advanced breast cancer (Rodriguez-Kraul et al, 1981; Tormey et al, 1983; Howell et al, 1984; Senn et al, 1984; Ang et al, 1989; Pronzato et al, 1989), however some retrospective studies have failed to demonstrate any significant correlation between dose and therapy efficacy (Ahmann et al, 1982; Glucksberg et al, 1982; Redmond et al, 1983; Mouridsen et al, 1984; Velez-Garcia et al, 1987). A series of analyses on published chemotherapy studies demonstrated that a positive correlation existed between the scheduled amount of drug per unit time and the efficacy of chemotherapy treatment on both early and advanced breast cancer (Hryniuk and Bush, 1984; Hryniuk and Levine, 1986). Only a few controlled prospective studies of the dose–response relationship in breast cancer have been published, and the results have not been consistent. A few studies have demonstrated a survival benefit from receiving a higher dose intensity (Carmo-Pereira et al, 1987; Tannock et al, 1988; Wood et al, 1994), while many controlled studies have failed to demonstrate any dose–response relationship (Hortobagyi et al, 1987*a*, *b*; Ludwig Breast Cancer Study Group, 1985; Walters et al, 1992; Fumoleau et al, 1993).

The retrospective calculation of dose-intensity has been criticized by Henderson et al (1988). In retrospective analyses, the correlation between higher dose and better therapy results could result from selection of patients with better tolerance to higher chemotherapy doses. Redmond et al (1983) have published an extensive review on the sources of error in retrospective correlations between dose intensity and outcome. As an illustration of the multiple sources of biases inherent in this kind of analysis, they demonstrated a significant correlation of dose intensity of placebo and outcome in two NSABP trials.

One way to circumvent these biases would be to correlate some biological measurement of dose intensity with treatment outcome. If the positive correlation between dose intensity and outcome were the result of selection bias, one would expect patients experiencing more toxicity to have the worse outcome, while the opposite should be true if the correlation results from a true dose–response effect. Perhaps the most important biological measure of drug effect is the haematological toxicity, which is the dose-limiting toxicity with most chemotherapy schedules.

The purpose of this study was to establish, through retrospective analyses, if a better prognosis was associated with patients having a lower leucocyte nadir (higher biological dose intensity) or with a higher leucocyte nadir (higher dose intensity because of better tolerance of chemotherapy). We also attempted to determine whether dose reductions of doxorubicin-containing adjuvant chemotherapy influenced the DDFS and OS of stage II and III breast cancer patients.

### PATIENTS AND METHODS

### Patients

The study population comprised 211 primary breast cancer patients who were included in two trials conducted simultaneously between 1981 and 1986 in Helsinki University Hospital, Department of Radiotherapy and Oncology. One hundred and forty-nine of the patients had stage II breast cancer and 62 stage III. All patients

*Received 22 February 1996*
*Revised 20 August 1996*
*Accepted 23 August 1996*

*Correspondence to:* C Blomqvist, Department of Oncology, Helsinki University Central Hospital, Haartmaninkatu 4, FIN-00290 Helsinki, Finland

**Table 1** Multivariate analyses of correlation between leucocyte count, initial dose intensities of doxorubicin and cyclophosphamide and DDFS and OS

| | DDFS | | | OS | | | No. of patients |
|---|---|---|---|---|---|---|---|
| Variable | Hazard ratio | (95% CI) | *P*-value | Hazard ratio | (95% CI) | *P*-value | |
| *Leucocyte nadir* | | | | | | | |
| Continuous | 1.42 | (1.13–1.78) | 0.006 | 1.32 | (1.03–1.68) | 0.04 | 193 |
| Categorical ($10^9$ $l^{-1}$ | | | | | | | |
| ≥4.0 | 1 | (reference) | | 1 | (reference) | | 11 |
| 3.0–3.9 | 1.03 | (0.45–2.50) | | 1.02 | (0.40–2.64) | | 35 |
| 2.0–2.9 | 0.57 | (0.25–1.34) | | 0.69 | (0.28–1.72) | | 81 |
| <2.0 | 0.37 | (0.15–0.91) | | 0.52 | (0.20–1.37) | | 66 |
| *Doxorubicin initial dose intensity* | | | | | | | |
| Continuous | 0.94 | (0.85–1.05) | 0.77 | 0.95 | (0.85–1.06) | 0.59 | 182 |
| Categorical (%) | | | | | | | |
| ≥90 | 1 | (reference) | | 1 | (reference) | | 48 |
| 80–89 | 0.83 | (0.45–1.52) | | 0.78 | (0.42–1.46) | | 46 |
| 70–79 | 1.08 | (0.56–2.07) | | 1.09 | (0.56–2.13) | | 56 |
| 60–69 | 1.21 | (0.51–2.85) | | 1.56 | (0.65–3.74) | | 18 |
| <60 | 0.79 | (0.20–3.19) | | 0.78 | (0.19–3.21) | | 14 |
| *Cyclophosphamide initial dose intensity* | | | | | | | |
| Continuous | 0.94 | (0.84–1.06) | 0.99 | 0.96 | (0.85–1.09) | 0.77 | 182 |
| Categorical (%) | | | | | | | |
| ≥90 | 1 | (reference) | | 1 | (reference) | | 37 |
| 80–89 | 1.51 | (0.80–2.84) | | 1.54 | (0.81–2.94) | | 71 |
| 70–79 | 1.32 | (0.60–2.93) | | 0.84 | (0.35–2.00) | | 40 |
| 60–69 | 1.26 | (0.51–3.07) | | 0.99 | (0.39–2.53) | | 22 |
| <60 | 3.03 | (0.70–13.04) | | 2.42 | (0.55–10.68) | | 12 |

Patients were stratified by stage (II *vs* III). The following variables were included: age (0–49, 50–59, 60 + years), oestrogen receptor status (negative < 10 ftmol, positive ≥ 10 ftmol), progesterone receptor status (negative < 10 ftmol, positive ≥ 10 ftmol), adjuvant treatment (chemotherapy only *vs* chemotherapy + radiotherapy *vs* chemotherapy + radiotherapy + tamoxifen). Age, ER, PgR, group, doxorubicin and cyclophosphamide dose intensity during the first two cycles had no significant effect on DDFS or OS.

underwent radical surgery and were treated with adjuvant chemotherapy. This consisted of eight 4-weekly cycles of cyclophosphamide (500 mg $m^{-2}$) and doxorubicin, (40 mg $m^{-2}$) administered intravenously on day 1, and ftorafur, an oral analogue of fluorouracil, (20 mg $kg^{-1}$) taken orally on days 1–14 (CAFt). Postoperative irradiation was given between the second and third adjuvant chemotherapy cycles using a cobolt source (45 Gy in 15 fractions) to regional nodes and operative scar. Adjuvant tamoxifen was given from the first day and continued for 2 years at 40 mg per day. Fifty-two stage II breast cancer patients were treated solely with chemotherapy (IIC), 47 with chemotherapy and radiotherapy (IIRC) and 50 with chemotherapy, radiotherapy and tamoxifen (IIRCT). Thirty-one stage III breast cancer patients were treated with chemotherapy and radiotherapy (IIIRC) and 31 with chemotherapy, radiotherapy and tamoxifen (IIIRCT). Details of these trials were published previously (Blomqvist et al, 1992; Saarto et al, 1995). No effect of tamoxifen or radiotherapy on distant disease-free survival or overall survival was demonstrated in either of these two trials. Routine blood counts were taken during every chemotherapy cycle, one day before treatment and approximately on day 10, in all patients during all chemotherapy cycles.

The mean age of the patients was 50.6 years. Oestrogen receptor values were available for 170 patients, 48% of whom were receptor positive. Progesterone receptor values were available for 171 patients, 45% of whom were receptor positive. One hundred and two (51%) patients were over 50 years old. Median follow-up time was 7.6 years.

### Definitions and calculation of dose intensity

Individual records were taken of the dosage of each chemotherapy drug, the duration of treatment and the body surface area. Absolute dose intensity was defined as the amount of drug administered per unit body surface area (mg $m^{-2}$) delivered per unit time (mg $m^{-2}$ $week^{-1}$). Relative dose intensity was calculated as delivered dose intensity divided by projected dose intensity, according to Longo et al (1991). The projected dose intensity is the total amount of drugs scheduled in the protocol, divided by the projected time schedule of the entire treatment. Delivered dose intensity represents the total amount of drug actually received, divided by the time taken for the therapy. Dose intensities for each drug were calculated for the total number of cycles (DI) and for the two initial cycles (DI2). Duration of treatment was defined as the interval (in weeks) between day one of the first cycle of chemotherapy and day 28 of the last given cycle.

For example, for a patient who received 450 mg $m^{-2}$ cyclophosphamide and 35 mg $m^{-2}$ doxorubicin (five cycles during 22 weeks), dose intensity of cyclophosphamide is [(450mg $m^{-2}$ × 5) /22 weeks]/125 mg $m^{-2}$ weeks = 0.818 = 81.8% and dose intensity of doxorubicin is [(35 mg $m^{-2}$ × 5)/22 weeks]/10 mg $m^{-2}$ week = 0.795 = 79.5%.

### Statistical methods

Five-year cumulative DDFS and OS rates were calculated using the Kaplan–Meier method (Kaplan and Meier, 1958). Multivariate

 © Cancer Research Campaign 1997

**Figure 1** Proportion with distant disease-free survival according to haematological toxicity in stage II and III patients. The times of censoring are depicted with vertical bars analyses were performed using the Cox regression technique (Cox, 1972) in EGRET software (SERC, 1988). Adjusted estimates were obtained from a model containing age at diagnosis (0–49, 50–59, 60–70 years), oestrogen receptors (positive, negative and missing), progesterone receptors (positive, negative and missing) and additional adjuvant therapy (none, radiotherapy, tamoxifen). Data for ftorafur were excluded from analyses because they had no statistically significant effect on therapy results in either preliminary univariate or multivariate analyses, and, furthermore, the data for ftorafur dose intensity were missing for 32 patients (data not shown). In the multivariate analysis, statistical significance was determined on the basis of likelihood ratio tests. All tests were two-sided and $P$-values less than or equal to 0.05 were considered statistically significant.

Of the 211 eligible patients, data from eleven patients were excluded from the analyses because they had not received the planned chemotherapy (five patients refused chemotherapy, four patients were not eligible for doxorubicin based chemotherapy because they were suffering from other severe diseases, one patient was excluded because of a non-radical operation and another because of haematogenous metastases), and a further six were excluded because they received only one cycle of chemotherapy ($n$=194). In the analyses of prognostic effect of leucocyte, there was one missing value ($n$=193). In analyses of dose intensity from the two initial cycles of chemotherapy, five patients had missing information on drug doses ($n$=189), and in analyses of total dose and dose intensity for all cycles, information of cumulative dose was missing in four patients ($n$=190). Overall dose intensity, total dose and efficacy of haematological toxicity were also analysed by excluding all patients who had received less than four cycles of chemotherapy ($n$=14) or whose treatment was interrupted because of progression during chemotherapy ($n$=8). No patients experienced disease progression during the first two cycles of therapy.

### RESULTS

Forty-nine patients (26%) discontinued chemotherapy as a result of side-effects, mostly nausea and vomiting. The median number of chemotherapy cycles was seven. Mean total dose of doxorubicin was 240.7 mg $m^{-2}$ (range 24–400 mg $m^{-2}$, 86% of the planned total dose. Mean total dose of cyclophosphamide was 2983.5 mg $m^{-2}$ (range 366–4114 mg $m^{-2}$), 75% of the planned total dose. Mean dose intensity of doxorubicin was 70% (range 21.6–109.9%) and of cyclophosphamide 70% (range 21.1–100%). Mean dose intensity of doxorubicin during the first two cycles was 82% (39.3–125%), and for cyclophosphamide it was also 82% (42–102.9%). The low level of dose intensity was partly as a result of unscheduled initial dose reductions of approximately 10%, which was later corrected by dose escalation at subsequent courses according to tolerance. Later dose reductions were mostly owing to leucopenia but, in some cases, also because of gastrointestinal side-effects.

In univariate analyses of DDFS and OS in the patients who have received at least two cycles of chemotherapy, a statistically significant correlation between DDFS or OS and the nadir values of leucocytes were established ($P$ = 0.01 and $P$ = 0.04 respectively). Patients with lower leucocyte nadirs had significantly better DDFS and OS than those patients with higher leucocyte nadir.

Doxorubicin dose intensity during the first two cycles of chemotherapy correlated significantly to DDFS when all patients received at least two cycles of chemotherapy were included ($P$=0.05). The total dose of doxorubicin and cyclophosphamide also correlated significantly to DDFS ($P$ = 0.01 and $P$ = 0.01 respectively) and OS ($P$ = 0.01 and $P$ = 0.01 respectively). Dose intensity of all cycles had no significant correlation to chemotherapy outcome.

After exclusion of patients who relapsed during the chemotherapy or discontinued chemotherapy because of toxicity before having received at least four cycles, a statistically significant correlation between DDFS and leucocyte nadir was still established ($P$ = 0.05). However, total dose of doxorubicin and cyclophosphamide were no longer significantly correlated, and dose intensity of all cycles, as before, had no significant effect on treatment outcome.

For multivariate analyses, age, hormonal receptor status, tamoxifen and radiotherapy treatment, delay between surgery and start of chemotherapy were included in the Cox model, although none of these variables had a statistically significant effect on outcome in univariate analyses. All patients who had received at least two cycles of chemotherapy were included in the analyses. In the multivariate analysis, leucocyte nadir still correlated significantly with DDFS and OS ($P$ = 0.006 and $P$ = 0.04 respectively), while doxorubicin and cyclophosphamide dose intensities of the first two

© Cancer Research Campaign 1997 

cycles lost their significance (Table 1). Similar correlation of leucocyte nadir with DDFS and OS was seen in multivariate analyses of stage II patients only ($P = 0.007$ and $P = 0.058$ respectively) (Figure 1).

Median DDFS and OS for leucocyte nadir ≥ 4.0, 3–3.9 and < 3.0 ($10^9$ $l^{-1}$ were 80 and 116, 90 and 111, and 126 and 128 months respectively.

### DISCUSSION

The interpretation of the dose–effect relationship in retrospective studies is problematic. Redmond et al (1983) was able to demonstrate, in retrospective analyses of 2 years' adjuvant chemotherapy, a significant dose–response effect even in the placebo group because of patients who discontinued therapy as a result of early recurrence or therapy toxicity. Geller et al (1990) also demonstrated that differences in disease-free survival (DFS) among patients in different chemotherapy dose regimens could be attributed to early recurrence. Our own results support these findings. The significant correlation between total dose and chemotherapy outcome was lost after excluding early failures who received lower doses because of treatment discontinuation.

One way of overcoming selection bias is to use only the initial dose intensity, as this might be less influenced by patient tolerance and is not affected by subsequent treatment discontinuation. In this study, we established a positive correlation between doxorubicin dose intensity during the first two cycles and distant disease-free survival (DDFS) No statistically significant correlation was found between dose intensity of all cycles and outcome. In contrast to our finding in a previous study, Hryniuk and Levine (1986) demonstrated significant correlation between dose intensity of all cycles and adjuvant chemotherapy outcome. However, the dose intensity was calculated with the minimal duration of therapy (at least six cycles of chemotherapy), and this automatically included early failures in the low-dose intensity group.

An even better way of overcoming selection bias is to correlate treatment outcome with some biological measurement of dose intensity, such as haematological toxicity which is perhaps the most straightforward. The correlation between lower leucocyte nadir and better outcome in the present study support a true dose–response relationship. In fact, a few previous studies of early and advanced breast cancer have also demonstrated a relationship between lower leucocyte nadirs and either a higher response rate or better DFS or OS (Ahmann et al, 1982; Samonigg et al, 1991; Yosef et al, 1993).

The results of the present study indicate that leucocyte nadir is an easy and reliable biological marker of chemotherapy efficacy. Breast cancer adjuvant chemotherapy with fixed protocol doses seemed to leave a group of patients who had better haematological tolerance with ineffective levels of dose intensity. More individual planning of chemotherapy doses is required. Using leucocyte nadir as a marker for therapy efficacy provides the possibility of establishing an optimal chemotherapy dose intensity for individual patients. The dose intensity during the initial cycles of chemotherapy, in particular, seems to be significant in the optimization of adjuvant chemotherapy.

### ACKNOWLEDGEMENT

This investigation was supported by grants awarded by the Radiological Society of Finland.

### REFERENCES

Ahmann DL, O'Fallon JR, Scanlon PW, Payne WS, Bisel HF, Edmonson JH, Frytak S, Hahn RG, Ingle JN, Rubin J and Creagan ET (1982) A preliminary assessment of factors associated with recurrent disease in a surgical adjuvant clinical trial for patients with breast cancer with special emphasis on the aggressiveness of therapy. *Am J Clin Oncol* **5**: 371–381

Ang PT, Buzdar AU, Smith TL, Kau S and Hortobagyi GN (1989) Analysis of dose intensity in doxorubicin-containing adjuvant chemotherapy in stage II and III breast carcinoma. *J Clin Oncol* **7**: 1677–1684

Blomqvist C, Tiusanen K, Elomaa I, Rissanen P, Hietanen T, Heinonen E and Grohn P (1992) The combination of radiotherapy, adjuvant chemotherapy (cyclophosphamide-doxorubicin-ftorafur) and tamoxifen in stage II breast cancer. Long-term follow-up results of a randomised trial. *Br J Cancer* **66**: 1171–1176

Bonadonna G and Valagussa P (1981) Dose response effect of adjuvant chemotherapy in breast cancer. *N Engl J Med* **34**: 10–15

Carmo-Pereira J, Costa FO, Henriques E, Godinho F, Cantinho LM, Sales LA and Rubens RD (1987) A comparison of two doses of adriamycin in the primary chemotherapy of disseminated breast carcinoma. *Br J Cancer* **56**: 471–473

Cox DR (1972) Regression models and life-tables. *JR Statist Soc Ser B* **34**: 187–220

Fisher B, Brown AM, Dimitrov NV, Poisson R, Redmond C, Margolese RG, Bowman D, Wolmark N, Wickerham DL, Kardinal CG, Shibata H, Paterson AHG, Sutherland CM, Robert NJ, Ager PJ, Levy L, Wolter J, Wozniak T, Fisher ER and Deutsch M (1990) Two months of doxorubicin-cyclophosphamide with and without interval reinduction therapy compared with 6 months of cyclophosphamide, methotrexate, and fluorouracil in positive-node breast cancer patients with tamoxifen-nonresponsive tumors: results from the National Surgical Adjuvant Breast and Bowel Project B-15. *J Clin Oncol* **8**: 1483–1496

Fumoleau P, Devaux Y, Vo Van ML, Kerbrat P, Fargeot P, Schraub S, Mihura J, Namer M and Mercier M (1993) Premenopausal patients with node-positive resectable breast cancer: preliminary results of a randomised trial comparing 3 adjuvant regimens: FEC 50 × 6 cycles vs FEC 50 × 3 cycles vs FEC 75 × 3 cycles. *Drugs* **45**: 38–45

Geller NL, Hakes TB, Petroni GR, Currie V and Kaufman R (1990) Association of disease-free survival and percent of ideal dose in adjuvant breast chemotherapy. *Cancer* **66**: 1678–1684

Glucksberg H, Rivkin SE, Rasmussen S, Tranum B, Gad Emn, Costanzi J, Hoogstraten B, Athens J, Maloney T, Mccracken J and Vaughn C (1982) Combination chemotherapy (CMFVP) versus L-phenylalanine mustard (L-PAM) for operable breast cancer with positive axillary nodes: a Southwest Oncology Group Study. *Cancer* **50**: 423–434

Henderson IC, Gelman RS, Harris JR and Canellos GP (1986) Duration of therapy in adjuvant chemotherapy trials. *Natl Cancer Inst Monogr* **1**: 95–98

Henderson IC, Hayes DF and Gelman R (1988) Dose-response in the treatment of breast cancer: a critical review. *J Clin Oncol* **6**: 1501–1515

Hortobagyi GN, Bodey GP, Buzdar AU, Frye D, Legha SS, Malik R, Smith TL, Blumenschein GR, Yap HY and Rodriguez V (1987*a*) Evaluation of high-dose versus standard FAC chemotherapy for advanced breast cancer in protected environment units: a prospective randomized study. *J Clin Oncol* **5**: 354–364

Hortobagyi GN, Buzdar AU, Bodey GP, Kau S, Rodriguez V, Legha SS, Yap HY and Blumenschein GR (1987*b*) High-dose induction chemotherapy of metastatic breast cancer in protected environment: a prospective randomized study. *J Clin Oncol* **5**: 178–184

Howell A, Rubens RD, Bush H, George WD, Howat JM, Crowther D, Sellwood RA, Hayward JL, Knight RK, Bulbrook RD, Fentiman IS and Chaudary M (1984) A controlled trial of adjuvant chemotherapy with melphalan versus cyclophosphamide, methotrexate, and fluorouracil for breast cancer. *Recent Results Cancer Res* **96**: 74–89

Hryniuk W and Bush H (1984) The importance of dose intensity in chemotherapy of metastatic breast cancer. *J Clin Oncol* **2**: 1281–1288

Hryniuk W and Levine MN (1986) Analysis of dose intensity for adjuvant chemotherapy trials in stage II breast cancer. *J Clin Oncol* **4**: 1162–1170

Kaplan EL and Meier P (1958) Nonparametric estimation from incomplete observations. *J Am Statist Assoc* **53**: 457–481

Longo DL, Duffey PL, Devita VJ, Wesley MN, Hubbard SM and Young RC (1991) The calculation of actual or received dose intensity: a comparison of published methods. *J Clin Oncol* **9**: 2042–2051

Ludwig Breast Cancer Study Group (1985) A randomized trial of adjuvant combination chemotherapy with or without prednisone in premenopausal breast cancer patients with metastases in one to three axillary lymph nodes. *Cancer Res* **45**: 4454–4459

© Cancer Research Campaign 1997

Mouridsen HT, Rose C, Brincker H, Thorpe SM, Rank F, Fischerman K and Andersen KW (1984) Adjuvant systemic therapy in high-risk breast cancer: the Danish Breast Cancer Cooperative Group's trials of cyclophosphamide or CMF in premenopausal and tamoxifen in postmenopausal patients. *Recent Results Cancer Res* **96**: 117–128

Pronzato P, Campora E, Amoroso D, Bertelli G, Botto F, Conte PF, Sertoli MR and Rosso R (1989) Impact of administration-related factors on outcome of adjuvant chemotherapy for primary breast cancer. *Am J Clin Oncol* **12**: 481–485

Redmond C, Fisher B and Wieand HS (1983) The methodologic dilemma in retrospectively correlating the amount of chemotherapy received in adjuvant therapy protocols with disease-free survival. *Cancer Treat Rep* **67**: 519–526

Rodriguez-Kraul R, Hortobagyi GN, Buzdar AU and Blumenschein GR (1981) Combination chemotherapy for breast cancer metastatic to bone marrow. *Cancer* **48**: 227–232

Saarto T, Blomqvist C, Tiusanen K, Gröhn P, Rissanen P and Elomaa I (1995) The prognosis of stage III breast cancer treated with postoperative radiotherapy and adriamycin-based chemotherapy with and without tamoxifen. Eight year follow-up results of a randomized trial. *Eur J Surg Oncol* **21**: 146–150

Samonigg H, Stoger H, Kasparek AK, Schmid M, Dusleag J, Pfeiffer K, Smola M, Steindorfer P and Lechner, P (1991) Prednimustine combined with mitoxantrone and 5-fluorouracil for first and second-line chemotherapy in advanced breast cancer. *Cancer Chemother Pharmacol* **27**: 477–480

Senn HJ, Jungi WF, Amgwerd R, Hochuli E, Ammann J, Engelhart G, Heinz C, Wick A, Enderlin F, Creux G, Simeon B, Lanz R, Bigler R and Seiler S (1984) Adjuvant chemoimmunotherapy with LMF + BCG in node-negative and node-positive breast cancer: 8 year results. *Recent Results Cancer Res* **96**: 90–101

SERC (1988) EGRET user's manual. Statistics and epidemiology research corporation: Seattle

Tancini G, Bonadonna G, Valagussa P, Marchini S and Veronesi U (1983) Adjuvant CMF in breast cancer: comparative 5-year results of 12 versus 6 cycles. *J Clin Oncol* **1**: 2–10

Tannock IF, Boyd NF, Deboer G, Erlichman C, Fine S, Larocque G, Mayers C, Perrault D and Sutherland H (1988) A randomized trial of two dose levels of cyclophosphamide, methotrexate, and fluorouracil chemotherapy for patients with metastatic breast cancer. *J Clin Oncol* **6**: 1377–1387

Tormey D, Gelman R and Falkson G (1983) Prospective evaluation of rotating chemotherapy in advanced breast cancer. An Eastern Cooperative Oncology Group Trial. *Am J Clin Oncol* **6**: 1–18

Velez-Garcia E, Carpenter JT, Moore M, Vogel CL, Marcial V, Ketcham A, Raney M and Smalley R (1987) Postsurgical adjuvant chemotherapy with or without radiotherapy in women with breast cancer and positive axillary nodes: progress report of a Southeastern Cancer Study Group (SEG) trial. *Adjuvant Ther Cancer* **5**: 347–355

Walters RS, Frye D, Buzdar AU, Holmes FA and Hortobagyi GN (1992) A randomized trial of two dosage schedules of mitomycin C in advanced breast carcinoma. *Cancer* **69**: 476–481

Wood WC, Budman DR, Korzun AH, Cooper MR, Younger J, Hart RD, Moore A, Ellerton JA, Norton L, Ferree CR, Ballow AC, Frei III E and Henderson IC (1994) Dose and dose intensity of adjuvant chemotherapy for stage II, node-positive breast carcinoma. *N Engl J Med* **330**: 1253–1259

Yosef H, Slater A, Keen CW, Bunting JS, Hope-Stone H, Parmar, H, Roberts JT, Termander B and Nilsson B (1993) Prednimustine (Sterecyt) versus cyclophosphamide both in combination with methotrexate and 5-fluorouracil in the treatment of advanced breast cancer. *Eur J Cancer* **29(A)**: 1100–1105

© Cancer Research Campaign 1997